# The therapeutic effects of physical activity on children with attention deficit hyperactivity disorder: A systematic review and meta-analysis

**DOI:** 10.1097/MD.0000000000042063

**Published:** 2025-04-18

**Authors:** Ruijuan Zheng, Silu Huang, Jianquan Yang, Pengju Zhao, Enyao Li

**Affiliations:** a Department of Children Rehabilitation Medicine, The Fifth Affiliated Hospital of Zhengzhou University, Henan Province, Zhengzhou, China.

**Keywords:** attention deficit hyperactivity disorder, children, meta-analysis, physical activity

## Abstract

**Background:**

Attention deficit hyperactivity disorder (ADHD) is a prevalent neurodevelopmental disorder in children, characterized by symptoms of inattention, hyperactivity, and impulsivity. Traditional treatments include pharmacological and behavioral therapies, which may have limitations. Physical activity has emerged as a potential non-pharmacological intervention for ADHD. This systematic review and meta-analysis aimed to evaluate the therapeutic effects of physical activity on ADHD symptoms, dropout rates, and social impairment in children.

**Methods:**

Adhering to the PRISMA guidelines, 4 electronic databases (PubMed, Embase, Web of Science, and Cochrane Library) were searched on July 18, 2024, without time or language restrictions. Randomized controlled trials involving children diagnosed with ADHD and examining the effects of physical activity interventions were included. Data extraction and quality assessment were independently conducted by 2 reviewers. Heterogeneity was assessed using chi-square statistics and the *I*^2^ value, and both fixed- and random-effects models were employed as appropriate. Sensitivity analysis and publication bias were also evaluated.

**Results:**

Out of 1103 articles initially identified, 7 studies met the inclusion criteria. The meta-analysis showed that physical activity significantly alleviated ADHD symptoms (Hedges’ g = ‐0.37, 95% CI [‐0.72, ‐0.02], *I*^2^ = 45.2%). Physical activity did not significantly affect dropout rates (Hedges’ g = 0.44, 95% CI [‐0.33, 1.2], *I*^2^ = 0.0%). However, it significantly reduced social impairment (Hedges’ g = ‐0.54, 95% CI [‐0.98, ‐0.10], *I*^2^ = 0.0%). Sensitivity analysis confirmed the stability and robustness of these findings. Funnel plots indicated no significant publication bias.

**Conclusion:**

Physical activity is an effective alternative treatment for ADHD, improving core symptoms and social impairment without affecting dropout rates. Incorporating physical activity into comprehensive ADHD management plans can enhance the overall well-being and quality of life for children with ADHD.

## 1. Introduction

Attention deficit hyperactivity disorder (ADHD) is a common neurodevelopmental disorder in children, marked by primary symptoms of inattention, hyperactivity, and impulsivity.^[1,2]^ These symptoms considerably hinder academic achievement, familial relationships, and social interactions, resulting in enduring functional and emotional repercussions. ADHD also imposes significant societal and economic challenges, influencing educational and occupational success. Conventional treatment modalities, including pharmacotherapy (e.g., stimulant medications) and behavioral therapies, are prevalent; however, they frequently encounter limitations due to side effects, adherence difficulties, and the necessity for ongoing clinical support. As a result, there is an increasing interest in investigating alternative non-pharmacological interventions, such as physical activity, to effectively mitigate ADHD symptoms.^[3,4]^

In recent years, physical activity has surfaced as a viable non-pharmacological strategy for ADHD, with increasing data endorsing its advantageous effects on symptom management.^[5,6]^ Research indicates that physical activity might augment cognitive performance, modulate neurotransmitter activity, and boost self-regulation, hence mitigating fundamental ADHD symptoms.^[7,8]^ Diverse modalities of physical activity, such as aerobic workouts, resistance training, and movement-oriented activities, provide adaptable alternatives for tailored interventions, accommodating human preferences and fitness levels. The variety of exercise modalities enables customized strategies that can enhance engagement and therapeutic results for children with ADHD. Notwithstanding increasing evidence, current studies on physical activity for ADHD are constrained by constraints, including small sample numbers, inconsistent study methodologies, and a restricted variety of interventions. Moreover, research has primarily concentrated on particular exercise modalities or subgroups, resulting in considerable information deficiencies. Adequate large-scale, systematic studies are lacking, and the possible differential impacts of physical exercise across age groups, genders, and ADHD subtypes (e.g., inattentive vs mixed type) remain insufficiently investigated.

This study posits that physical activity serves as an effective non-pharmacological intervention for mitigating fundamental ADHD symptoms, such as inattention, hyperactivity, and impulsivity, while concurrently enhancing social functioning in children. The principal objective of this research is to methodically assess and integrate the effects of physical activity on these outcomes via a meta-analysis. This study aims to address deficiencies in the current literature by offering substantial, impartial, and thorough evidence about the therapeutic benefits of physical activity for children with ADHD. This research will elucidate the efficacy and practicality of physical activity, thereby providing useful insights for the development of integrative therapy options in ADHD management.

## 2. Methods

### 2.1. Search strategy

During the systematic review process, adherence to the Preferred Reporting Items for Systematic Reviews and Meta-Analyses (PRISMA) guidelines was maintained.^[9]^ Four electronic databases—PubMed, Embase, Web of Science, and Cochrane Library—were searched on July 18, 2024, without applying any time restrictions. The search strategy employed was: (“Attention Deficit Hyperactivity Disorder” or “ADHD”) and (“Children” or “Pediatric”) and (“Physical Activity” or “Exercise” or “Aerobic Exercise” or “Resistance Training”) and (“Therapeutic Effects” or “Treatment” or “Intervention”). No language limitations were applied. Additionally, the reference lists of relevant articles were manually screened for any additional potential records.

### 2.2. Inclusion criteria and exclusion criteria

Inclusion criteria:

(1) Population: Studies that included children diagnosed with ADHD.
(2) Intervention: Studies examining the effects of physical activity interventions, including but not limited to aerobic exercises, resistance training, and mind–body practices such as yoga and tai chi.
(3) Outcomes: Studies reporting on outcomes related to ADHD symptoms, such as attention, hyperactivity, and impulsivity, as well as secondary outcomes like academic performance and social functioning.
(4) Study design: Only randomized controlled trials were included.

Exclusion criteria:

(1) Population: Studies that included adults or populations without a formal ADHD diagnosis.
(2) Intervention: Studies that did not specifically examine physical activity as an intervention or combined physical activity with other interventions in a way that did not allow for separate analysis.
(3) Outcomes: Studies that did not report relevant outcomes related to ADHD symptoms or secondary outcomes of interest.
(4) Accessibility: Studies for which full-text articles were not available.

### 2.3. Data extraction

Two evaluators separately conducted literature screening and data extraction, subsequently cross-verifying the results. In the event of inconsistencies during this procedure, the reviewers engaged in discussions to resolve the issues, potentially consulting a third-party reviewer if required. The gathered data comprised the author(s) of the study, publication year, number of cases analyzed, study design, participant age and gender, diagnosis, and specifics of the physical activity intervention (length, frequency, duration, and intensity). In the absence of pertinent data in the published report, we reached out to the original study investigators via email to solicit the unpublished data.

### 2.4. Quality assessment

The quality of the included studies was assessed utilizing the Cochrane Collaboration’s risk of bias tool.^[10]^ Two reviewers separately evaluated 6 domains: random sequence generation, allocation concealment, blinding of participants and personnel, inadequate outcome data, selective reporting, and additional potential sources of bias. Each domain was assessed as low, ambiguous, or high risk of bias. In instances of discord, the reviewers deliberated to achieve consensus or sought the opinion of a third reviewer when required. This meticulous examination guaranteed a comprehensive and impartial appraisal of the study quality for the meta-analysis.

### 2.5. Statistical analyses

The variability among research was evaluated using chi-square statistics and measured by the *I*^2^ score. When the *I*^2^ value was below 50% and the accompanying *P*-value was larger than or equal to .10, it signified the absence of significant heterogeneity, prompting the use of the fixed-effect model to calculate the aggregated effect size. In contrast, when the *I*^2^ value was 50% or above, or the associated *P*-value was below .10, significant heterogeneity was indicated, prompting the adoption of the random-effects model to compute the combined effect size. A sensitivity analysis was performed to assess the robustness of the results and to determine the potential impact of individual studies on the overall effect size. This entailed the systematic exclusion of each research from the meta-analysis and the subsequent recalibration of the overall effect magnitude. Furthermore, the symmetry of the funnel plot was examined to identify any possible publishing bias. A balanced distribution of data points at the apex of the funnel plot would indicate a less probability of publication bias affecting the meta-analysis outcomes. All statistical tests were bilateral, and a *P*-value below .05 was considered statistically significant. Data were analyzed utilizing Stata version 17 (StataCorp, College Station, TX).

## 3. Results

### 3.1. Search results and study selection

**Figure 1.**
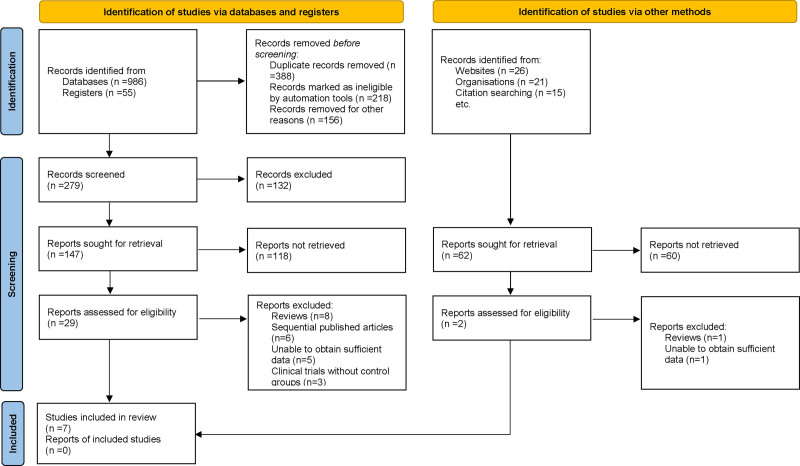
Flow diagram of the study selection process for included studies.

During the preliminary phase of this systematic review and meta-analysis, a comprehensive search of multiple electronic databases produced 1103 possibly pertinent publications. An algorithm was utilized to eliminate duplicate entries from the dataset, guaranteeing that each distinct investigation was represented singularly. Titles and abstracts were thoroughly assessed according to strictly established inclusion and exclusion criteria, encompassing study methodology, demographic attributes of the study population, measured clinical outcomes, and the overall quality of research methodologies. Subsequent to this initial filtration, 31 articles were selected for further examination. Numerous researchers independently scrutinized the complete text of each paper to guarantee an impartial and thorough evaluation. In this phase, 24 papers were removed for the following reasons: review articles (n = 9), consecutively published works (n = 6), inadequate data for analysis (n = 6), and clinical trials without control groups (n = 3). As a result, 7 publications fulfilled all rigorous criteria outlined in our research process and were incorporated into the final meta-analysis^[11–17]^ (Fig. 1).

### 3.2. Study characteristics

The included studies investigated various physical activity interventions for children with ADHD, encompassing a range of exercise modalities and formats. The sample sizes of the studies varied from 18 to 112 patients, with the mean ages ranging from approximately 6.6 to 15.9 years. The majority of participants were male, reflecting a common demographic trend in ADHD studies. Interventions included exergaming, high-intensity exercises, horseback riding, motor skill practice, table tennis, aerobic exercises, and goal-directed activities, often facilitated by professional trainers and tailored to individual or group settings. The duration of each session varied from 10 to 60 minutes, with frequencies ranging from 2 to 3 times per week over periods of 6 to 12 weeks (Table 1).

**Table 1:** Summary of physical activity interventions in children with ADHD.

Authors	Year	Patients	Age (M ± SD)	Gender	Diagnosis	Intervention content	Length (minutes)	Frequency (weekly)	Duration (weeks)
Benzing et al	2019	51	10.43 ± 1.37	82% male	ADHD	Exergaming (individual, parent)	30	3 times	8
Gelade et al	2018	112	6.63 ± 1.76	75% male	ADHD	Warm-up, cool down, high-intensity exercise at 70%–80% of HRmax, high-intensity exercise at 80%–100% of HRmax (individual, investigator)	10	3 times	10–12
Oh et al	2018	34	8.15 ± 1.57	91% male	ADHD	Horseback riding + unmounted activities (group-based, professional trainer)	60	2 times	12
Garcia-Gomez et al	2016	18	10.49 ± 1.78	67% male	ADHD	Horseback riding + unmounted activities (group-based, professional trainer)	45	2 times	12
Pan et al	2016	32	8.9 ± 1.5	100% male	ADHD	Warm-up, cool down, motor skill practice, executive function focused table tennis exercise (individual, professional trainer)	10, 20, 40	2 times	12
Choi et al	2015	35	15.9 ± 1.2	100% male	ADHD	Stretching, feedback and cooling down, aerobic exercise (running, jumping rope, basketball) (group, professional trainers)	20, 60	3 times	6
Kang et al	2011	32	8.49 ± 1.03	100% male	ADHD	Goal-directed activities (e.g., darts), shuttle runs, jump roping (group-based, professional trainer)	20, 15, 20	2 times	6

ADHD = attention deficit hyperactivity disorder, HRmax = maximum heart rate.

### 3.3. Results of quality assessment

**Figure 2.**
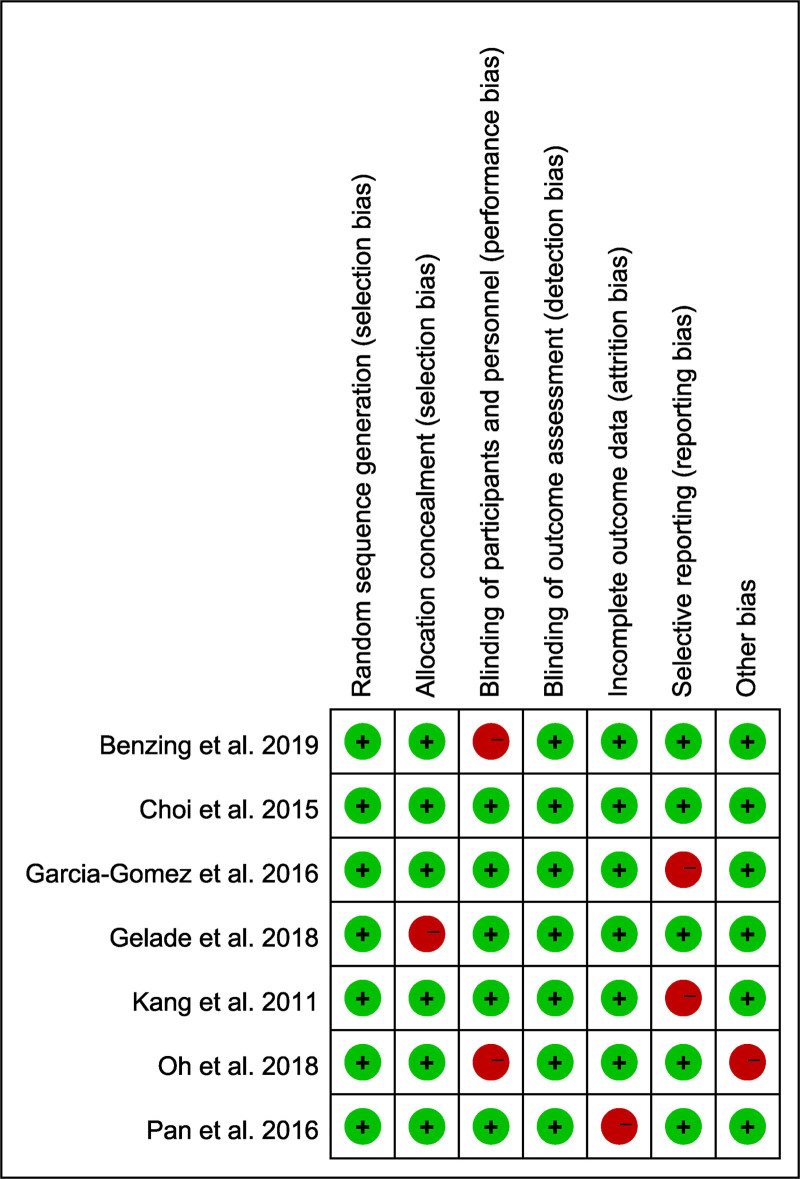
Quality assessment of included studies using the Cochrane Collaboration’s risk of bias tool. Red indicates high risk, green indicates low risk.

The quality assessment of the included studies revealed a generally low risk of bias across most domains. Random sequence generation and allocation concealment were rated as low risk in the majority of studies, indicating robust randomization and allocation procedures. However, blinding of participants and personnel was identified as a potential source of performance bias in several studies, with a few studies showing high or unclear risk in this domain. Blinding of outcome assessment was consistently rated as low risk, ensuring objective measurement of outcomes. Incomplete outcome data and selective reporting were also rated as low risk in most studies, suggesting that attrition and reporting biases were minimal. A few studies exhibited unclear or high risk for other potential sources of bias, but these were not widespread. Overall, the methodological quality of the included studies was deemed acceptable for the meta-analysis, with most studies demonstrating rigorous adherence to standard research protocols (Fig. 2).

### 3.4. Therapeutic impact of physical activity on ADHD core symptoms in children

**Figure 3.**
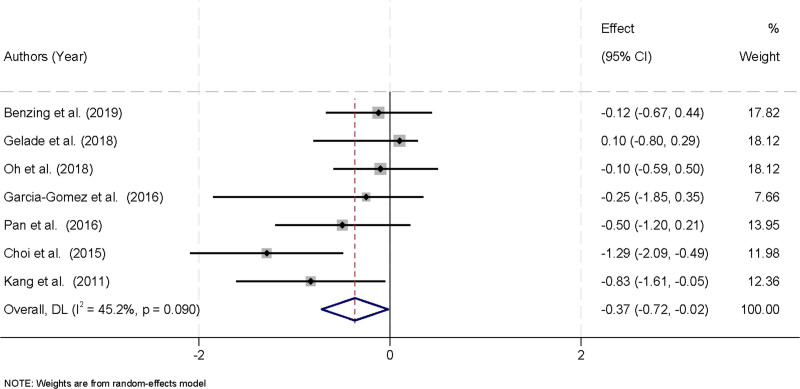
Forest plot illustrating the therapeutic impact of physical activity on core ADHD symptoms in children. ADHD = attention deficit hyperactivity disorder.

A total of 7 studies were included in the meta-analysis, all of which reported on the total ADHD core symptoms at posttreatment. There was notable heterogeneity among the studies (*I*^2^ = 45.2%, *P* = .09), prompting the use of a random-effects model to combine the results. The meta-analysis demonstrated that physical activity significantly alleviated ADHD symptoms in children, indicating a beneficial therapeutic effect. The pooled effect size, represented by Hedges’ g, was ‐0.37 with a 95% confidence interval of ‐0.72 to ‐0.02, showing statistical significance. This negative value of Hedges’ g suggests that the physical activity interventions led to a reduction in ADHD symptoms compared to control groups (Fig. 3). Overall, these findings support the efficacy of physical activity as a treatment strategy for ADHD in children, emphasizing its potential as a non-pharmacological intervention that can be incorporated into comprehensive ADHD management plans.

### 3.5. Impact of physical activity on study dropout rates in children with ADHD

**Figure 4.**
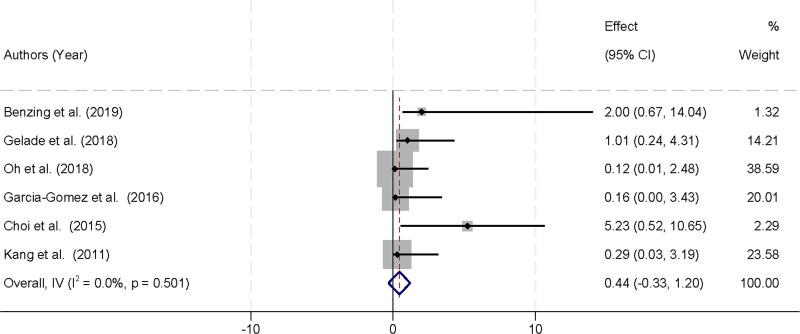
Forest plot illustrating the impact of physical activity on study dropout rates in children with ADHD. ADHD = attention deficit hyperactivity disorder.

Six studies were included in the meta-analysis, all of which reported dropout rates over the course of the study among children with ADHD undergoing physical activity interventions. The analysis revealed no significant heterogeneity among these studies (*I*^2^ = 0.0%, *P* = .501), which justified the use of a fixed-effects model to combine the results. The pooled data indicated that physical activity interventions did not have a statistically significant impact on dropout rates when compared to control conditions, with Hedges’ g calculated at 0.44 (95% CI [‐0.33, 1.2]) (Fig. 4). These findings suggest that physical activity does not significantly influence the likelihood of children with ADHD dropping out over the duration of the study. The confidence interval, while wide, indicates variability in individual study outcomes but shows an overall neutral effect of physical activity on dropout rates.

### 3.6. Effect of physical activity on social impairment in children with ADHD

**Figure 5.**
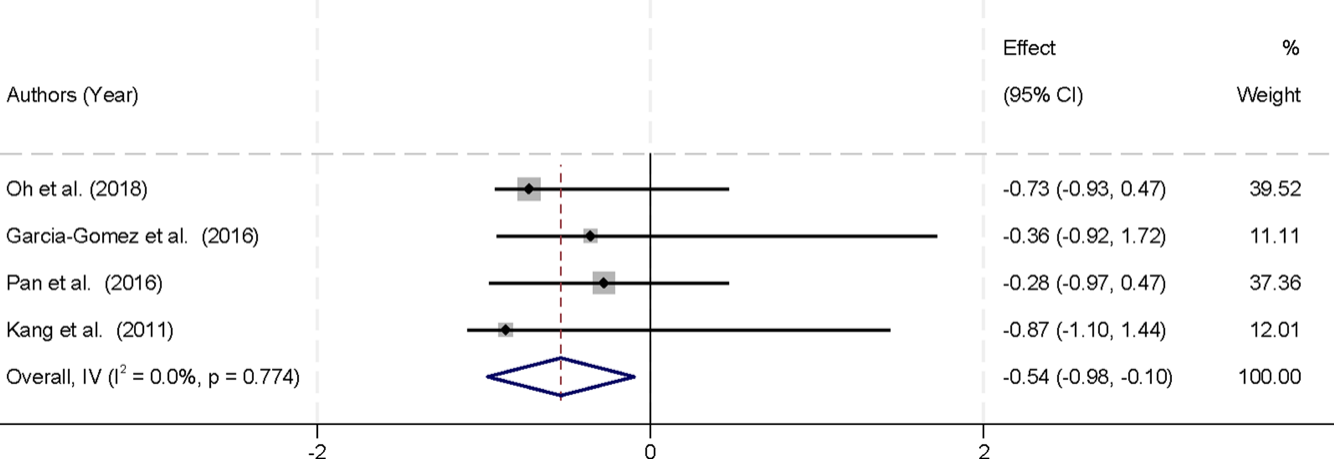
Forest plot illustrating the effect of physical activity on social impairment in children with ADHD. ADHD = attention deficit hyperactivity disorder.

Four studies were included in the meta-analysis, all of which assessed functional impairment in the social context among children with ADHD. The analysis showed no significant heterogeneity among these studies (*I*^2^ = 0.0%, *P* = .774), supporting the use of a fixed-effects model to combine the results. The pooled analysis revealed that physical activity interventions significantly alleviated social impairment in children with ADHD, with Hedges’ g calculated at ‐0.54 (95% CI [‐0.98, ‐0.10]) (Fig. 5). These findings indicate that physical activity has a significant positive impact on reducing social impairment in children with ADHD. The negative value of Hedges’ g suggests that children who participated in physical activity interventions experienced fewer social difficulties compared to those in the control group. This improvement in social functioning is crucial, as social impairment is a core challenge for children with ADHD, affecting their ability to interact with peers and form meaningful relationships. The results underscore the potential of physical activity as an effective intervention to address social impairment in children with ADHD, highlighting its role as a valuable component of comprehensive ADHD management plans. By improving social functioning, physical activity can contribute to better overall quality of life and enhanced social integration for children with ADHD.

### 3.7. Sensitivity analysis

**Figure 6.**
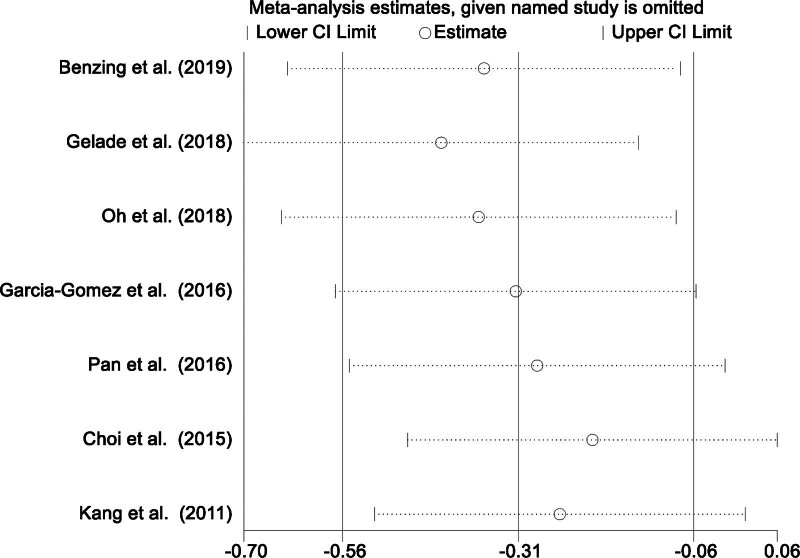
Sensitivity analysis of the therapeutic impact of physical activity on core ADHD symptoms in children. ADHD = attention deficit hyperactivity disorder.

Due to the notable heterogeneity observed among the studies assessing the therapeutic impact of physical activity on ADHD core symptoms, a sensitivity analysis was conducted to evaluate the stability and reliability of the pooled results. This analysis involved sequentially excluding each individual study and recalculating the combined effect estimates for the remaining studies. The sensitivity analysis demonstrated that the pooled results remained stable and robust, regardless of the exclusion of any single study. This finding indicates that no single study had an undue influence on the overall results, thereby enhancing the reliability of our conclusions. The consistency of the results across these analyses highlights the robustness of our primary findings and further supports the conclusions drawn from this meta-analysis (Fig. 6).

### 3.8. Publication bias

**Figure 7.**
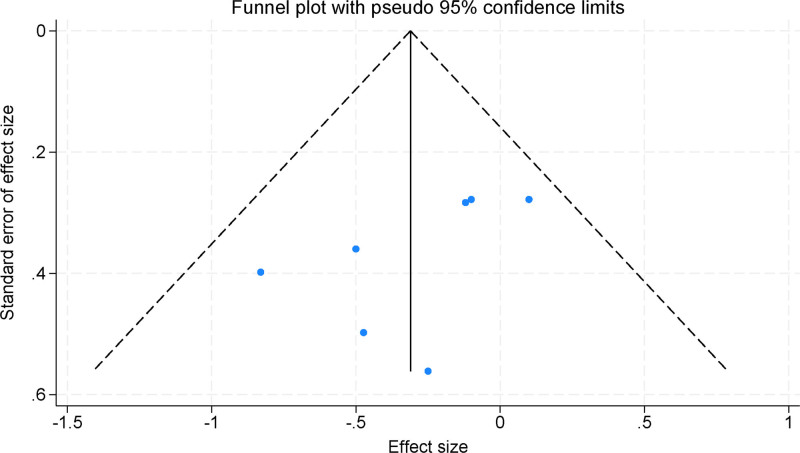
Funnel plot evaluating publication bias in the included studies.

The funnel plots constructed with the included studies demonstrated symmetry, indicating no significant publication bias (Fig. 7). This symmetrical distribution of data points suggests that the meta-analysis results were not influenced by selective publication, thus reinforcing the validity and reliability of the findings.

## 4. Discussion

ADHD is a prevalent neurodevelopmental disorder characterized by symptoms of inattention, hyperactivity, and impulsivity.^[18,19]^ Previous research has suggested that exercise can increase levels of neurotransmitters such as dopamine and norepinephrine, which are critical for attention and executive function.^[20,21]^ Tao et al^[22]^ reported that multicomponent and mind–body physical activity interventions yield cognitive improvements in children with neurodevelopmental disorders; our findings align by demonstrating significant amelioration of ADHD core symptoms and social impairment, with our focused analysis and rigorous sensitivity testing conferring superior methodological precision. Singh et al^[23]^ synthesized evidence across diverse populations demonstrating exercise-induced improvements in cognitive domains, particularly executive functions in ADHD; in concordance, our focused meta-analysis on children with ADHD confirms significant reductions in core symptoms and social impairment, while also addressing intervention acceptability through dropout analysis. Van Riper et al^[6]^ investigated the neurobiological mechanisms underlying exercise-induced cognitive changes in adolescents with ADHD, revealing altered brain activity patterns during dual-task performance. Their study provides mechanistic insights, demonstrating that exercise modulates neural activation in frontal and parietal regions associated with ADHD-related executive dysfunction. Our study complements these findings by confirming the therapeutic efficacy of physical activity in reducing ADHD symptoms and social impairment.

The examination of 7 studies indicated that physical activity markedly mitigates basic ADHD symptoms in youngsters. The effect size, denoted by Hedges’ g, was ‐0.37 (95% CI [‐0.72, ‐0.02]), signifying a moderate yet statistically significant decrease in ADHD symptoms after physical activity interventions. This discovery is significant as it highlights the possibility of physical activity to function as an adjuvant treatment for ADHD, supplementing conventional pharmaceutical and behavioral therapies. The factors that contribute to the positive effects of physical activity on ADHD symptoms may be complex. Physical activity is recognized to improve cognitive performance by elevating neurotransmitter levels, including dopamine and norepinephrine, which are essential for attention and executive function. Exercise-induced neuroplasticity may enhance cognitive performance by fostering the development of new brain connections and reinforcing existing ones.^[24,25]^ Moreover, physical activity can modulate arousal levels, alleviate stress, and enhance mood, all of which are elements that can beneficially affect attention and behavior in children with ADHD.

The analysis of dropout rates from 6 studies indicated that physical activity interventions did not significantly influence the likelihood of children with ADHD dropping out of the studies. The effect size, represented by Hedges’ g, was 0.44 (95% CI [‐0.33, 1.2]), and the analysis showed no significant heterogeneity (*I*^2^ = 0.0%, *P* = .501). This finding suggests that physical activity is generally well-tolerated by children with ADHD and does not lead to higher dropout rates, which is an important consideration for the feasibility of implementing physical activity programs in real-world settings. The neutral impact of physical activity on dropout rates may be attributed to several factors. Physical activities, especially those that are engaging and enjoyable, may enhance motivation and adherence among children. Furthermore, the structured and routine nature of physical activity programs can provide a sense of predictability and stability, which may be beneficial for children with ADHD.^[26,27]^ These programs also offer opportunities for social interaction and peer support, which can enhance the overall experience and reduce the likelihood of dropout.

The meta-analysis of 4 studies evaluating social impairment in children with ADHD demonstrated that physical activity substantially mitigates social challenges, with an impact size of ‐0.54 (95% CI [‐0.98, ‐0.10]). The analysis indicated no substantial heterogeneity (*I*^2^ = 0.0%, *P* = .774), implying uniform results across investigations. Social impairment is a significant obstacle for children with ADHD, hindering their capacity to establish and sustain connections. The notable decrease in social impairment after physical activity interventions underscores the capacity of these programs to enhance social functioning and overall quality of life for children with ADHD.^[28,29]^ Various processes may elucidate the beneficial effects of physical activity on social impairment. Physical activities, particularly those in group contexts, offer inherent chances for social contact, collaboration, and communication. These interactions facilitate the development of essential social skills in youngsters, including turn-taking, sharing, and adherence to norms.^[30,31]^ Furthermore, enhancements in mood and self-esteem derived from consistent physical activity can bolster social confidence and diminish social anxiety, so promoting favorable social interactions.

The assessment of heterogeneity and potential publication bias is crucial for ensuring the reliability and validity of meta-analysis findings. In this review, heterogeneity was evaluated using the *I*² statistic, which quantifies the proportion of variation attributable to heterogeneity rather than chance. For the core symptom analysis, an *I*² value of 45.2% indicated moderate heterogeneity, reflecting variability in study outcomes due to differences in intervention types, sample sizes, and methodological approaches. Consequently, a random-effects model was applied to account for these variations. Sensitivity analyses further confirmed the robustness of the findings, as the exclusion of individual studies did not significantly alter the overall effect estimates. Regarding publication bias, funnel plot analysis demonstrated no significant asymmetry, suggesting that selective publication was unlikely to have influenced the results. This enhances the validity and generalizability of the findings, reinforcing the therapeutic benefits of physical activity for ADHD symptoms. In contrast, analyses of dropout rates and social impairment exhibited no significant heterogeneity (*I*² = 0.0%), justifying the use of a fixed-effects model. Additionally, funnel plot inspection revealed no substantial asymmetry, further indicating the absence of publication bias. These findings collectively support the robustness and consistency of the therapeutic effects of physical activity on ADHD symptoms and social impairment, underscoring its potential as a non-pharmacological intervention.

The results of this meta-analysis have significant implications for the treatment and management of ADHD in children. Physical exercise interventions present a viable non-pharmacological strategy that can be incorporated into complete ADHD management strategies. Considering the moderate effect sizes indicated, physical activity need to be regarded as an adjuvant treatment rather than an independent therapy. Nonetheless, its prospective advantages, such as enhancements in primary symptoms and social functioning, render it a significant element of multimodal ADHD therapy approaches. This meta-analysis possesses multiple limitations. The variability in study designs, intervention types, and outcome measures may impact the generalizability of the findings. The studies considered exhibited variability in sample size and quality, which may have introduced bias. The dependence on published data may have led to publication bias. Ultimately, the brief duration of the majority of interventions constrains the comprehension of the long-term impacts of physical activity on ADHD symptoms. Future research should prolong the duration of therapies and incorporate long-term follow-up to thoroughly assess the enduring effects of physical activity on ADHD.

## 5. Conclusions

Physical activity proves to be a viable non-pharmacological intervention for ADHD, significantly reducing core symptoms (inattention, hyperactivity, and impulsivity) and social impairment in children. Notably, physical activity does not significantly affect study dropout rates, underscoring its feasibility in long-term interventions. These findings advocate for the integration of physical activity into comprehensive ADHD treatment strategies, demonstrating its potential to improve both symptom management and overall quality of life for children with ADHD. By offering an effective alternative to traditional treatments, physical activity can play a critical role in enhancing the therapeutic approach to ADHD.

## Acknowledgments

The research was funded by Natural Science Foundation of Henan Province (No. 212300410399) and the Collaborative Innovation Project of Zhengzhou (No. 18XTZX12003).

## Author contributions

**Conceptualization:** Ruijuan Zheng.

**Data curation:** Ruijuan Zheng, Jianquan Yang.

**Formal analysis:** Ruijuan Zheng, Silu Huang, Jianquan Yang.

**Investigation:** Ruijuan Zheng, Jianquan Yang.

**Methodology:** Ruijuan Zheng, Silu Huang, Jianquan Yang.

**Resources:** Silu Huang.

**Software:** Silu Huang.

**Supervision:** Pengju Zhao, Enyao Li.

**Writing – original draft:** Ruijuan Zheng.

**Writing – review & editing:** Pengju Zhao, Enyao Li.

## References

[1] Rajaprakash M Leppert ML. Attention-deficit/hyperactivity disorder. Pediatr Rev. 2022;43:135–47. 35229109 10.1542/pir.2020-000612

[2] Thapar A Cooper M. Attention deficit hyperactivity disorder. Lancet. 2016;387:1240–50. 26386541 10.1016/S0140-6736(15)00238-X

[3] Sharma A Couture J. A review of the pathophysiology, etiology, and treatment of attention-deficit hyperactivity disorder (ADHD). Ann Pharmacother. 2014;48:209–25. 24259638 10.1177/1060028013510699

[4] Cortese S Coghill D. Twenty years of research on attention-deficit/hyperactivity disorder (ADHD): looking back, looking forward. Evid Based Ment Health. 2018;21:173–6. 30301823 10.1136/ebmental-2018-300050 PMC10270437

[5] Chan YS Jang JT Ho CS. Effects of physical exercise on children with attention deficit hyperactivity disorder. Biomed J. 2022;45:265–70. 34856393 10.1016/j.bj.2021.11.011 PMC9250090

[6] Riper SMV Tempest GD Piccirilli A Ma Q Reiss AL. Aerobic exercise, cognitive performance, and brain activity in adolescents with attention-deficit/hyperactivity disorder. Med Sci Sports Exerc. 2023;55:1445–55. 36897828 10.1249/MSS.0000000000003159

[7] Memarmoghaddam M Torbati HT Sohrabi M Mashhadi A Kashi A. Effects of a selected exercise programon executive function of children with attention deficit hyperactivity disorder. J Med Life. 2016;9:373–9. 27928441 PMC5141397

[8] Russell D Arnold LE. Complementary and integrative treatments for attention-deficit/hyperactivity disorder in youth. Child Adolesc Psychiatr Clin N Am. 2023;32:173–92. 37147036 10.1016/j.chc.2022.08.005

[9] Page MJ McKenzie JE Bossuyt PM. The PRISMA 2020 statement: an updated guideline for reporting systematic reviews. BMJ. 2021;372:n71. 33782057 10.1136/bmj.n71 PMC8005924

[10] Higgins JP Altman DG Gøtzsche PC. The Cochrane Collaboration’s tool for assessing risk of bias in randomised trials. BMJ. 2011;343:d5928. 22008217 10.1136/bmj.d5928 PMC3196245

[11] Benzing V Schmidt M. The effect of exergaming on executive functions in children with ADHD: a randomized clinical trial. Scand J Med Sci Sports. 2019;29:1243–53. 31050851 10.1111/sms.13446

[12] Choi JW Han DH Kang KD Jung HY Renshaw PF. Aerobic exercise and attention deficit hyperactivity disorder: brain research. Med Sci Sports Exerc. 2015;47:33–9. 24824770 10.1249/MSS.0000000000000373 PMC5504911

[13] García-Gómez A Rodríguez-Jiménez M Guerrero-Barona E Rubio-Jiménez JC García-Peña I Moreno-Manso JM. Benefits of an experimental program of equestrian therapy for children with ADHD. Res Dev Disabil. 2016;59:176–85. 27614276 10.1016/j.ridd.2016.09.003

[14] Geladé K Janssen TWP Bink M. A 6-month follow-up of an RCT on behavioral and neurocognitive effects of neurofeedback in children with ADHD. Eur Child Adolesc Psychiatry. 2018;27:581–93. 29098467 10.1007/s00787-017-1072-1

[15] Kang KD Choi JW Kang SG Han DH. Sports therapy for attention, cognitions and sociality. Int J Sports Med. 2011;32:953–9. 22068930 10.1055/s-0031-1283175

[16] Oh Y Joung YS Jang B. Efficacy of hippotherapy versus pharmacotherapy in attention-deficit/hyperactivity disorder: a randomized clinical trial. J Altern Complement Med. 2018;24:463–71. 29641212 10.1089/acm.2017.0358

[17] Pan CY Chu CH Tsai CL Lo SY Cheng YW Liu YJ. A racket-sport intervention improves behavioral and cognitive performance in children with attention-deficit/hyperactivity disorder. Res Dev Disabil. 2016;57:1–10. 27344348 10.1016/j.ridd.2016.06.009

[18] Aranas K Leighton JP. Dimensions of physical activity as related to child attention-deficit/hyperactivity disorder symptoms and impairment. Clin Child Psychol Psychiatry. 2022;27:953–66. 34875896 10.1177/13591045211058338 PMC9574890

[19] Caponnetto P Casu M Amato M. The effects of physical exercise on mental health: from cognitive improvements to risk of addiction. Int J Environ Res Public Health. 2021;18:13384. 34948993 10.3390/ijerph182413384 PMC8705508

[20] Liang X Qiu H Wang P Sit CHP. The impacts of a combined exercise on executive function in children with ADHD: a randomized controlled trial. Scand J Med Sci Sports. 2022;32:1297–312. 35611615 10.1111/sms.14192

[21] Gapin JI Labban JD Etnier JL. The effects of physical activity on attention deficit hyperactivity disorder symptoms: the evidence. Prev Med. 2011;52(Suppl 1):S70–4. 21281664 10.1016/j.ypmed.2011.01.022

[22] Tao R Yang Y Wilson M Chang JR Liu C Sit CHP. Comparative effectiveness of physical activity interventions on cognitive functions in children and adolescents with neurodevelopmental disorders: a systematic review and network meta-analysis of randomized controlled trials. Int J Behav Nutr Phys Act. 2025;22:6. 39806448 10.1186/s12966-024-01702-7 PMC11731537

[23] Singh B Bennett H Miatke A. Effectiveness of exercise for improving cognition, memory and executive function: a systematic umbrella review and meta-meta-analysis [published online ahead of print March 6, 2025]. Br J Sports Med. doi: 10.1136/bjsports-2024-108589. 10.1136/bjsports-2024-108589 PMC12229068 40049759

[24] Tandon M Pergjika A. Attention deficit hyperactivity disorder in preschool-age children. Child Adolesc Psychiatr Clin N Am. 2017;26:523–38. 28577607 10.1016/j.chc.2017.02.007

[25] Zhang Z Li R Zhou Z Wang P Yang B Wang X. The effect of physical activity on quality of life and parenting stress in children with attention-deficit/hyperactivity disorder: a randomized controlled trial. Disabil Health J. 2023;16:101377. 36202733 10.1016/j.dhjo.2022.101377

[26] Rommel AS Lichtenstein P Rydell M. Is physical activity causally associated with symptoms of attention-deficit/hyperactivity disorder? J Am Acad Child Adolesc Psychiatry. 2015;54:565–70. 26088661 10.1016/j.jaac.2015.04.011 PMC4984951

[27] Ganjeh P Meyer T Hagmayer Y. Physical activity improves mental health in children and adolescents irrespective of the diagnosis of attention deficit hyperactivity disorder (ADHD)—a multi-wave analysis using data from the KiGGS Study. Int J Environ Res Public Health. 2021;18:2207. 33668090 10.3390/ijerph18052207 PMC7967688

[28] Miklós M Komáromy D Futó J Balázs J. Acute physical activity, executive function, and attention performance in children with attention-deficit hyperactivity disorder and typically developing children: an experimental study. Int J Environ Res Public Health. 2020;17:4071. 32517384 10.3390/ijerph17114071 PMC7312258

[29] Den Heijer AE Groen Y Tucha L. Sweat it out? The effects of physical exercise on cognition and behavior in children and adults with ADHD: a systematic literature review. J Neural Transm (Vienna). 2017;124(Suppl 1):3–26. 10.1007/s00702-016-1593-7 PMC5281644 27400928

[30] Barnard-Brak L Davis T Sulak T Brak V. The association between physical education and symptoms of attention deficit hyperactivity disorder. J Phys Act Health. 2011;8:964–70. 21885887 10.1123/jpah.8.7.964

[31] Taylor A Novo D Foreman D. An exercise program designed for children with attention deficit/hyperactivity disorder for use in school physical education: feasibility and utility. Healthcare (Basel). 2019;7:102. 31487932 10.3390/healthcare7030102 PMC6787573

